# 

**Published:** 1883-02

**Authors:** 


					﻿ESTABLISHED IN 1855.
C>Li> OriiTE*?	TrUPTTAPV 1GftQ	New Serie
Vol. XX—No. 11.	X-iS-DXVUaxVI , lOOO.	Vol. Il-No. 6
1 1 ___________
ATLANTA
MEDICAL REGISTER.
{ATLANTA MEDICAL AND SURGICAL JOURNAL.)
ZEZDiarszD
JAMES B. BAIRD, M.D.
H.H. DICKSON, PUBLISHER. TERMS. $2-50 A YEAR IN ADVANCE.
ATLANTA, GEORGIA:
H. JI. D1CKE0N, BOOK ARD JOB IR1KTER ARD BOOK BIRDER.
				

